# Establishment of optimal exercise therapy using near-infrared spectroscopy monitoring of tissue muscle oxygenation after therapeutic angiogenesis for patients with critical limb ischemia: A multicenter, randomized, controlled trial

**DOI:** 10.1016/j.conctc.2020.100542

**Published:** 2020-02-04

**Authors:** Keisuke Shoji, Kenji Yanishi, Hirokazu Shiraishi, Shiho Yamabata, Arito Yukawa, Satoshi Teramukai, Kojiro Imai, Toshiko Ito-Ihara, Masami Tao, Yukihito Higashi, Tomoaki Ishigami, Yoshihiro Fukumoto, Koichiro Kuwahara, Satoaki Matoba

**Affiliations:** a Department of Cardiovascular Medicine, Kyoto Prefectural University of Medicine, Kyoto, Japan; b Rehabilitation Unit, University Hospital, Kyoto Prefectural University of Medicine, Kyoto, Japan; c Department of Biostatistics, Graduate School of Medical Science, Kyoto Prefectural University of Medicine, Kyoto, Japan; d Department for Medical Innovation and Translational Medical Science, Kyoto Prefectural University of Medicine Graduate School of Medical Science, Kyoto, Japan; e The Clinical and Translational Research Center, University Hospital, Kyoto Prefectural University of Medicine, Kyoto, Japan; f Department of Cardiovascular Regeneration and Medicine, Research Institute for Radiation Biology and Medicine, Hiroshima University, Hiroshima, Japan; g Department of Stem Cell and Immune Regulation, Yokohama City University Graduate School of Medicine, Kanagawa, Japan; h Department of Internal Medicine, Division of Cardiovascular Medicine, Kurume University School of Medicine, Fukuoka, Japan; i Department of Cardiovascular Medicine, Shinshu University School of Medicine, Nagano, Japan

**Keywords:** Critical limb ischemia, Arteriosclerosis obliterans, Therapeutic angiogenesis, Optimal exercise therapy, Tissue muscle oxygen saturation, Near-infrared spectroscopy, CLI, critical limb ischemia, PAD, peripheral artery disease, ASO, arteriosclerosis obliterans, BM-MNC, bone marrow-derived mononuclear cells, TAO, thromboangiitis obliterans, NO, nitric oxide, eNOS, endothelial nitric oxide synthase, VAS, visual analogue scale, CT, computed tomography, TcPO_2_, transcutaneous oxygen pressure, SPP, skin perfusion pressure, WIQ, walking impairment questionnaire, RHI, reactive hyperemia index, StO_2_, thenar tissue oxygen saturation, NIRS, near-infrared spectroscopy, TOI, tissue oxygenation index, nTHI, normalized tissue hemoglobin index, ΔO_2_Hb, change in oxygenated hemoglobin concentration, ΔHHb, change in deoxygenated hemoglobin concentration

## Abstract

Critical limb ischemia (CLI) is a potentially life-threatening condition that involves severely reduced blood flow to the peripheral arteries due to arteriosclerosis obliterans (ASO) of the limbs or a similar condition. CLI patients must undergo revascularization to avoid amputation of the lower limbs and improve their survival prognosis. However, the outcomes of conventional surgical revascularization or endovascular therapy are inadequate; therefore, establishing further effective treatment methods is an urgent task. We perform therapeutic angiogenesis using autologous bone marrow-derived mononuclear cells in clinical practice and demonstrated its safety and efficacy for CLI patients for whom conventional treatments failed or are not indicated. Exercise therapies must be devised for CLI patients who have undergone therapeutic angiogenesis to save their limbs and improve survival. Because evidence regarding the efficacy and safety of exercise therapy for CLI patients is lacking, we plan to perform a prospective trial of the efficacy and safety of optimal exercise therapy following therapeutic angiogenesis for CLI patients.

The trial will enroll 30 patients between 20 and 79 years with Rutherford category 4 or 5 CLI caused by ASO who will undergo therapeutic angiogenesis. Participants will be randomly allocated to receive either optimal exercise therapy or fixed exercise therapy. Those receiving optimal exercise therapy will undergo tissue muscle oxygen saturation monitoring using near-infrared spectroscopy while performing exercises and will be prescribed optimal exercise therapy. The optimal amount of exercise will be determined on day 8, 31, 61, 91 and 181 after therapeutic angiogenesis.

**Ethics and dissemination:**

This protocol was approved by the Institutional Review Boards of Kyoto Prefectural University of Medicine. In accordance with the Helsinki Declaration, written informed consent has been obtained from all participants prior to enrollment. The results of this trial will be disseminated by publication in a peer-reviewed journal.

**Trial registration:**

This trial is registered at http://www.umin.ac.jp/ctr/index.htm (identifier: UMIN000035288).

## 1 Introduction

Critical limb ischemia (CLI) is the end stage of peripheral artery disease (PAD), which occurs when peripheral artery stenosis or occlusion severely reduces blood flow, thereby causing the appearance of resting pain, gangrene and ulceration of the limbs. Patients with lifestyle-related diseases, older individuals, and those undergoing hemodialysis are at high risk. However, the underlying cause varies widely, with diseases including arteriosclerosis obliterans (ASO), thromboangiitis obliterans (TAO), and vasculitis due to collagen diseases eventually leading to CLI. The yearly major limb amputation rate for CLI is approximately 30%; in approximately 20% of cases, the condition reportedly persists despite successful revascularization or other standard treatment [1]. The survival prognosis is extremely poor, with 1-year survival rates of 70%–80% and 5-year survival rates of 50% [1]. Cardiovascular events are one of the important prognostic factor in CLI patients. Thus, CLI leads to a high rate of limb amputation and is potentially fatal. Moreover, as its prevalence increases with age, the number of patients with CLI associated with ASO is also expected to increase rapidly. Therefore, the establishment of further methods of treatment other than the current standard therapies is an urgent task. Therapeutic angiogenesis using autologous bone marrow-derived mononuclear cells (BM-MNC) had been undertaken in clinical practice and demonstrated its safety and efficacy for no-option CLI patients caused by ASO, TAO, and vasculitis due to collagen diseases for whom conventional treatments failed or are not indicated [[2], [3], [4], [5], [6], [7]]. However, the long-term survival rate and limb salvage rate were lower in CLI patients associated with ASO after therapeutic angiogenesis compared to those associated with TAO or collagen diseases [7]. Therefor it is necessary to further improve the survival rate and limb salvage rate in CLI and ASO patients.

Although exercise therapy for ASO patients with claudication is recommended in several guidelines, the therapy for CLI and ASO patients is not recommended from the view point of worsening of limb ischemia and deterioration of their wound [1,8]. However, exercise therapy can be introduced after therapeutic angiogenesis, and may provide the effect of preventing physical function deterioration and progression of lower limb ischemia and arteriosclerosis. Therefore, we should establish appropriate exercise intensity and frequency that do not lead deterioration of lower extremity ischemia and wound for CLI patients after therapeutic angiogenesis. Herein, we have planned a clinical trial to identify the direct efficacy of appropriate exercise therapy for limb ischemia for patients who have undergone therapeutic angiogenesis.

### 1.1 Endpoints

The primary endpoint is the change in the maximum walking distance 6 months after therapeutic angiogenesis (day 181) compared with baseline. The secondary endpoints are changes in the following parameters day 181 compared with baseline: pain-free walking distance; resting pain measured using the Visual Analogue Scale (VAS), on which 0 cm indicated pain-free or no pain and 10 cm indicated the most severe pain; skeletal muscle mass of the ischemic limb measured by a body composition analysis and lower limb contrast-enhanced computed tomography (CT); transcutaneous oxygen pressure (TcPO_2_) and skin perfusion pressure (SPP) of a dorsal or plantar region in an affected lower limb; walking impairment questionnaire (WIQ) scale; Fontaine class and Rutherford category; reactive hyperemia index (RHI) score (vascular endothelial function); muscle strength (grip strength and isometric knee extension strength); vascular volume below the knee of the ischemic limb measured by lower limb contrast-enhanced CT; number of ischemic ulcers on the ischemic limb; and maximum area (length × width) of ischemic ulceration on the ischemic limb.

Safety endpoints were the incidence, severity, and seriousness of adverse events; overall survival duration; incidence of major limb amputation; and time to major amputation.

## 2 Materials and methods

### 2.1 Study design

This is a multicenter, open-label, randomized, controlled trial.

### 2.2 Study setting

Five hospitals have agreed to participate in this trial. This protocol (protocol number: CQARD-CAR-180701) has been approved by the Institutional Review Boards of Kyoto Prefectural University of Medicine. In accordance with the Helsinki Declaration, written informed consent has been obtained from all participants prior to enrollment.

### 2.3 Participants

The inclusion criteria are as follows: patients registered for therapeutic angiogenesis using autologous bone marrow-derived mononuclear cells (BM-MNC) implantation conducted as a clinical trial under the Act on the Safety of Regenerative Medicine; CLI patients with Rutherford category 4 or 5; patients capable of undergoing near-infrared spectroscopy (NIRS) measurements at the specified sites; and patients of either sex between 20 and 79 years who have provided written consent to participate in the trial based on the will of the patients themselves and/or the understanding and approval of their families after having received a full explanation of the advantages and disadvantages arising from exercise therapy and the advantages and disadvantages arising from not undergoing exercise therapy by means of an informed consent form.

The exclusion criteria are as follows: patients who do not provide informed consent or who are unsuitable for the trial because of emotional considerations, even if the disease and procedure are suitable; remaining life expectancy of less than 1 year because of another comorbid condition; untreated limb with Fontaine class III or worse; undergoing treatment for active malignancy; ischemic heart disease that has not been treated with revascularization; untreated severe diabetic retinopathy; serious infection; serious hepatic impairment or serious renal impairment (excluding chronic maintenance dialysis patients); serious hematological disorder such as leukopenia or thrombocytopenia or severe anemia necessitating blood transfusion; pregnancy, potential pregnancy, or breastfeeding; other severe acute or chronic medical or psychiatric conditions or abnormal clinical test results for which participation in the trial may result in increased risk or that may affect the interpretation of the trial results; unable to engage fully in exercise therapy due to cerebrovascular diseases, limb amputation, or other diseases; and deemed unsuitable to participate in the trial by an investigator or sub-investigator for any other reason.

### 2.4 Randomization

**Fig. 1:**
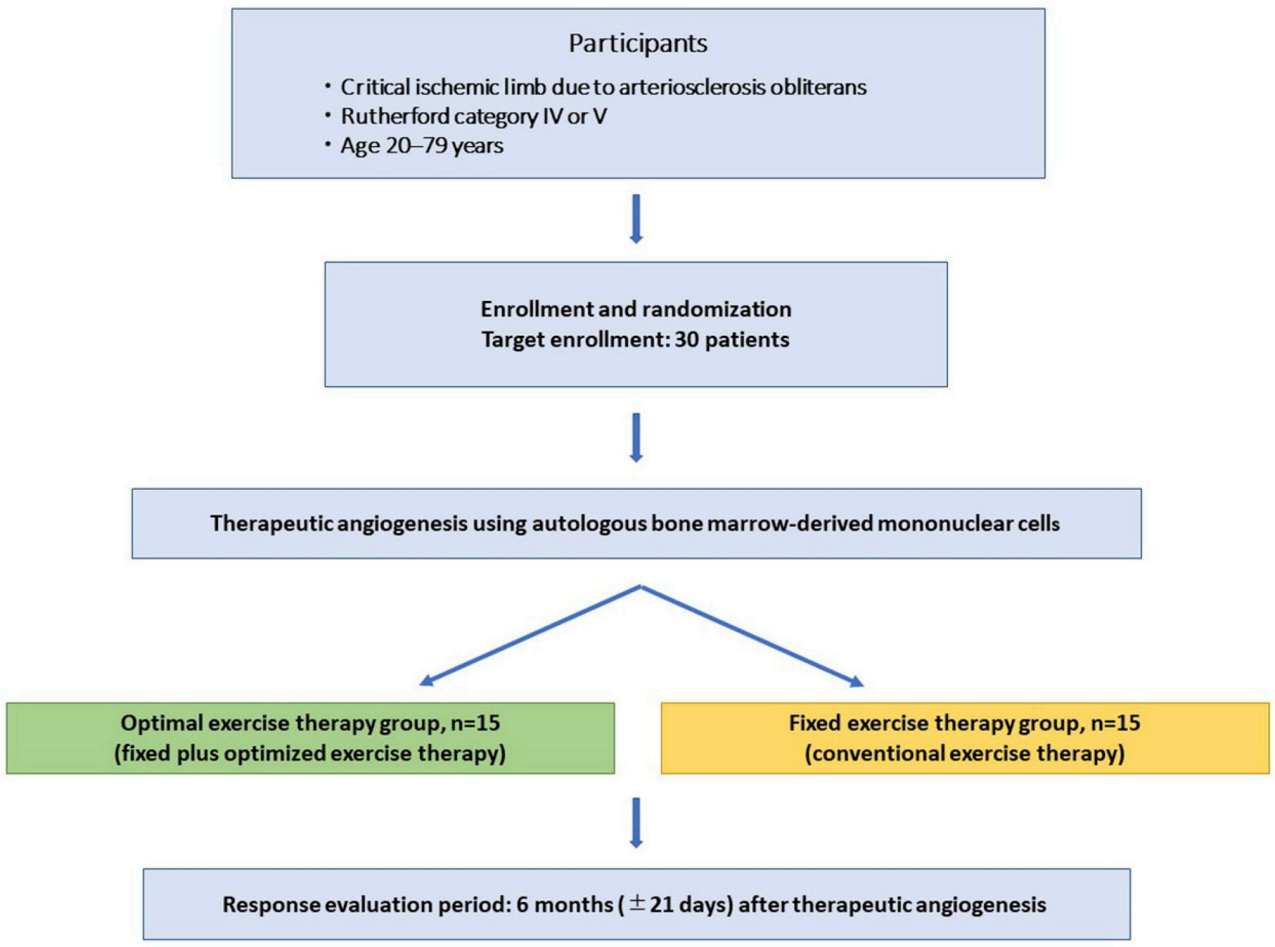
Study flow chart.

Patient enrollment and randomization will be performed at the central data center of Kyoto Prefectural University of Medicine. The eligible participants will be randomly allocated in a 1:1 ratio using a truncated stratified permuted block randomization with stratification for institution to receive either optimal exercise therapy or fixed exercise therapy (Fig. 1).

### 2.5 Treatment regimens and hemodynamic evaluation of the ischemic limb

#### 2.5.1 Evaluation

1. Maximum walking distance and Pain-free walking distance

Patients walk quickly on a flat surface, and the maximum walking distance (m) of which they are capable despite leg symptoms (such as claudication and pain) will be measured. Pain-free walking distance (the maximum distance they can walk without leg symptoms) will also be measured at the same time.

2. Treadmill walking test monitoring tissue muscle oxygenation by NIRS

In this trial, we use NIRS (NIRO-200NX; Hamamatsu Photonics Co., Ltd.) to evaluate tissue muscle oxygenation in the target limb. During the treadmill walking test, we continuously evaluate by NIRS oxygenated hemoglobin concentration (ΔO_2_Hb), change in deoxygenated hemoglobin concentration (ΔHHb), a tissue oxygenation index (TOI) and normalized tissue hemoglobin index (nTHI) which are indicators of tissue muscle oxygenation. The NIRS fiber-optic probe and light detector will be attached to the anterior tibialis and lateral gastrocnemius muscles of the treated leg. Measurements will be started 1 min before the treadmill walking test with standing position, and ΔO_2_Hb, ΔHHb, and variations in nTHI and TOI will be measured over time at a sampling frequency of 1 Hz. After the end of the treadmill walking test, the recording will be continued until dissociation between ΔO_2_Hb, ΔHHb and nTHI converges.

Following the partly modified method of Gardner et al. [9], exercise intensity will start at a speed of 0.5 km/h and increase after 1 min in 0.5-km/h increments up to a maximum of 5.0 km/h; then, the patient will continue to walk at this rate (5.0 km/h) (maximum 10 min). The treadmill walking test will be ended if symptom limitations** are reached. Then, the patient will remain seated at rest until the ΔO_2_Hb, ΔHHb, and nTHI recover and symptoms improve, and the time from the end of the treadmill walking test until recovery from symptoms (symptom recovery time) will be measured.

**Reasons for ending the test are defined as severe leg symptoms (such as claudication or pain), chest pain, unbearable dyspnea, leg spasm, unsteadiness, excessive sweating, facial pallor or cyanosis, or TOI <30% with a low nTHI.

#### 2.5.2 Exercise protocol

We divided into 2 groups: optimal exercise therapy group and fixed exercise therapy group.

#### 2.5.3 Fundamental exercise

We will start fundamental exercise without load on the ischemic wound (consisted of warming up, leg stretches and resistance training) from day 2 after therapeutic angiogenesis and continue until the end of the protocol in both groups.

#### 2.5.4 Exercise protocol in the optimal exercise therapy group

**Fig. 2:**
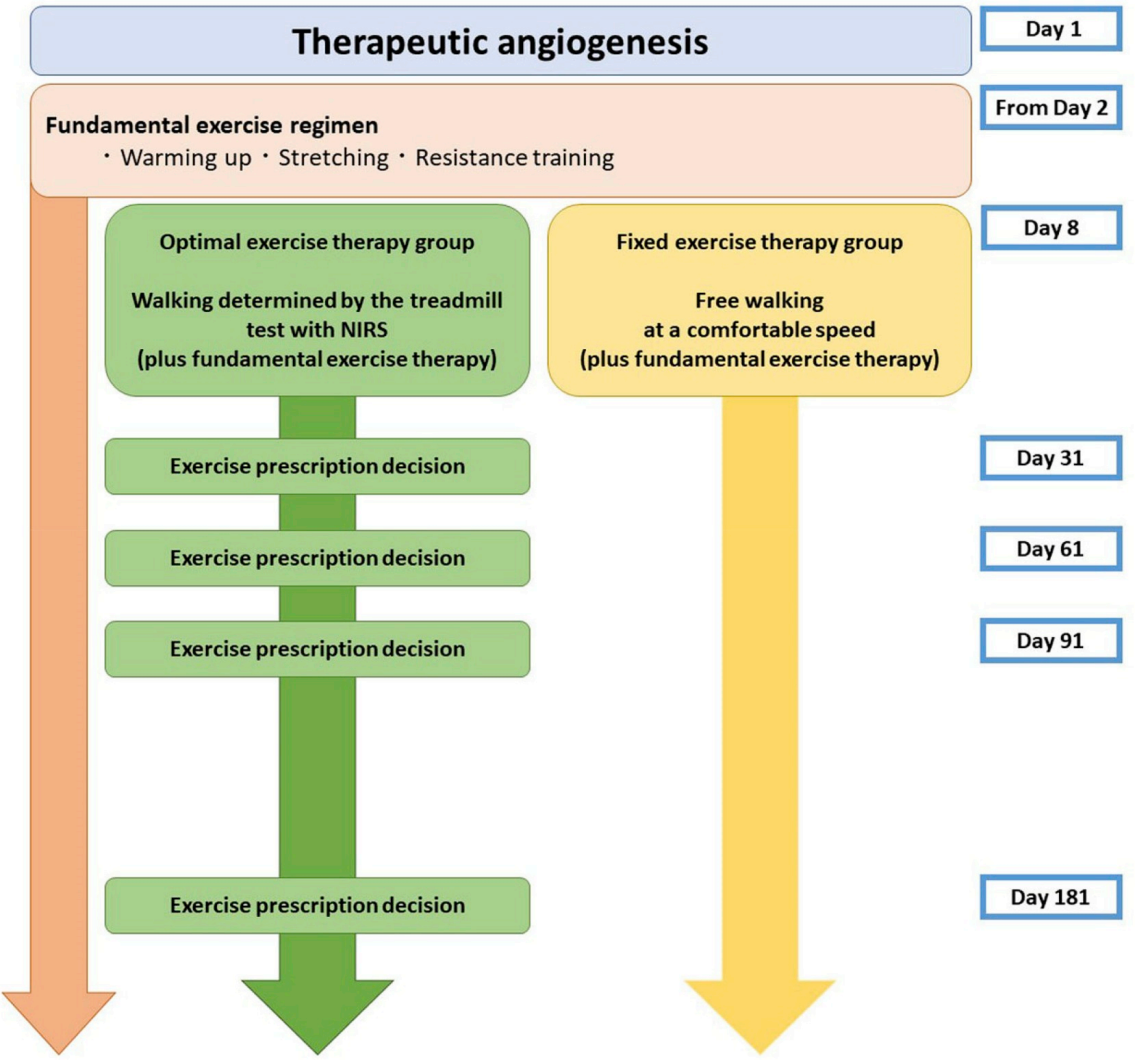
Manual of exercise therapy for each group. NIRS, near-infrared spectroscopy.

From day 8, optimal exercise therapy which consists of Treadmill walking will be started in addition to the fundamental exercise (Fig. 2). Based on the treadmill walking test and hemodynamic evaluation by NIRS, exercise at a walking speed of 80% of the walking speed at the end of the treadmill test will be prescribed as the exercise regimen. During the protocol therapy period, patients of the optimal exercise therapy group are instructed to walk at this exercise intensity according to this regimen for at least 30 min and at least three times a week (home-based exercise). Exercise regimen will be re-evaluated by treadmill test with using NIRS on day 8, 31, 61, 91 and 181 after therapeutic angiogenesis. (Fig. 2).

#### 2.5.5 Exercise protocol in the fixed exercise therapy group

In addition to the fundamental exercise (Fig. 2), the patients in the fixed exercise therapy group will be allowed free walking for disuse syndrome preventing from day 8 and will be given exercise instructions to continue walking on the comfortable speed before discharge. Subsequently, they will visit attend outpatient appointments a hospital at the same timing points as those used for the optimal exercise therapy group (day 31, 61, 91, and 181 after therapeutic angiogenesis); again, they will receive the same type of instruction.

Optimal exercise therapy will be implemented as follows. First, the maximum walking distance will be measured, and the level of challenge on the treadmill walking test will be adjusted accordingly. During the treadmill walking test, patients will undergo tissue muscle oxygenation monitoring with NIRS.

#### 2.5.6 Exercise management during the protocol therapy period

In both group, the amount of exercise or daily activities performed during the protocol therapy period will be monitored with an Actiwatch (Philips, N.V., USA), which generates a signal corresponding to the level of movement and its frequency by the accelerometer and records the movement as an activity count. Participants of the optimal exercise therapy group will receive instructions regarding exercise approximately once per week. During the protocol therapy period, record books will be issued, and either the patients themselves or a family member will record their daily amounts of exercise and activity levels. The attending physician will check these during study period to hospital visits by the participants and evaluate the compliance of exercise therapy.

### 2.6 Rationale for the sample size

In a trial that evaluated improvements in the walking ability of patients with arteriosclerosis obliterans for which revascularization is infeasible (Rutherford category 1–3) as a result of exercise therapy following Gardner's modified protocol [9], which is the same protocol used in our trial, the maximum walking distance in the exercise therapy intervention group improved from 143 ± 68 m before intervention to 452 ± 177 m after intervention. Based on the study by Kim et al. [10], we considered that because the participants of this trial had Rutherford category 4 or 5, the improvements in their walking ability would be inferior to that of patients in that study; therefore, we set the anticipated value of the improvement in maximum walking distance as 280 m. Based on a study by Matoba et al. [2], that evaluated walking distance 6 months after therapeutic angiogenesis, the anticipated improvement in maximum walking distance in the fixed exercise therapy group was set at 100 m. To achieve ≥80% power using a *t*-test with a one-sided significance level of 0.05, assuming a standard deviation of 160 m for both groups, the required sample size in each group is 11. To account for participants excluded from the analysis sets, the target sample size was set at 15 patients per group (total of 30 patients in both groups).

### 2.7 Data management

Data management will be performed by the Clinical and Translational Research Center, University Hospital, Kyoto Prefectural University of Medicine. To ensure the reliability and proper management of all study-related data, the data management director and manager will conduct quality-control procedures in each stage of the data handling process based on the data management protocol.

### 2.8 Statistical considerations

The safety analysis set will comprise all those enrolled patients who underwent the protocol therapy. The efficacy analysis set will comprise patients in the safety analysis population other than those found to be ineligible after enrollment. For the primary endpoint, the change (difference) in the maximum walking distance 6 months after therapeutic angiogenesis compared with baseline between the two groups will be compared using a one-sided *t*-test. For the secondary endpoints, the following parameters will be analyzed using a *t*-test: change (difference) in pain-free walking distance (the distance walked by the patient before leg pain starts), Visual Analogue Scale (VAS) score, skeletal muscle mass of the ischemic limb measured by body composition analysis and lower limb contrast-enhanced CT, TcPO_2_, SPP, WIQ scale, RHI score (vascular endothelial function), and muscle strength (grip strength, isometric knee extension strength), vascular volume below the knee of the ischemic limb measured by lower limb contrast-enhanced CT, the number of ischemic ulcers on the ischemic limb, and the maximum area (length × width [cm^2^]) of ischemic ulceration on the ischemic limb. For changes in the Fontaine class and Rutherford category, the differences between the two groups before and after therapy will be compared using the Wilcoxon rank sum test.

For safety endpoints, the occurrence, frequency, and severity of all adverse events occurring during the trial will be summarized for the safety analysis set. The time from therapeutic angiogenesis to either major amputation or death from any cause will be used to estimate amputation-free survival rates for the two groups by the Kaplan-Meier method. A similar analysis will be conducted for overall mortality using the time from therapeutic angiogenesis to death from any cause.

### 2.9 Ethics and dissemination

This trial received ethical approval from the Institutional Review Board of Kyoto Prefectural University of Medicine (approval number: ERB-C-1377). It is subject to Institutional Review Board supervision and control. This trial is registered at http://www.umin.ac.jp/ctr/index.htm (identifier: UMIN000035288). The results of this trial will be disseminated by publication in a peer-reviewed journal.

### 2.10 Trial status

The enrollment period for this trial is from the date of notification of Institutional Review Board approval until March 31, 2021. The protocol therapy period is 6 months after therapeutic angiogenesis (day 181) ±21 days, and the trial period is from the date of notification of Institutional Review Board approval until March 31, 2022.

## 3 Discussions

We perform therapeutic angiogenesis using autologous BM-MNC as an advanced medical treatment in our clinical practice, and its efficacy and safety for patients with CLI have been published in several reports [[2], [3], [4], [5], [6], [7]]. Therapeutic angiogenesis consists of extracting the mononuclear cell fraction, which contains immature cells that will differentiate into vascular endothelium (vascular endothelial precursor cells), from autologous bone marrow and injecting these into skeletal muscle exhibiting ischemic symptoms to generate new blood vessels. After the Therapeutic Angiogenesis Using Cell Transplantation (TACT) study reported the angiogenic effects of BM-MNC in CLI in 2002, this method was translated to an advanced medical treatment and is now used in clinical practice [3]. Since then, several follow-up studies and randomized trials have found that therapeutic angiogenesis significantly improves the rates of survival and limb salvage compared with the conventional standard therapies, and they have confirmed its efficacy for improving resting pain and extending pain-free walking distances [2,[4], [5], [6]]. Recent study following the prognosis of CLI patients after therapeutic angiogenesis reported good long-term clinical outcomes (The 10-year overall survival rate was 46.6% in patients with ASO, 90.5% in patients with TAO, and 67.6% in patients with collagen disease. The 10-year major amputation free rate was 70.1%, 87.9%, and 90.9%, respectively) [7]. The clinical outcomes in CLI patients after BM-MNC implantation in this study were not inferior to those after conventional treatment in previous studies. Considering this study enrolled the CLI patients who were intractable to any other conventional treatment options, BM-MNC implantation might be as effective or better than conventional treatments. CLI is a disease with an extremely poor prognosis for survival and for the lower limb, and its incidence is expected to increase in the future as society ages. Because the number of patients with refractory CLI is projected to increase, it will be necessary to save the limbs of even more patients and further improve their survival. Therapeutic angiogenesis using autologous BM-MNC implantation has improved the limb salvage rate for patients with CLI due to ASO who have not responded to conventional treatments or whose conditions are not indicated for conventional treatment, but it is still insufficient. New treatment options are required to improve limb salvage and survival rates among patients with no-option CLI who have undergone therapeutic angiogenesis.

Exercise therapy interventions for ASO patients with moderate and severe claudication for which revascularization is infeasible have been shown to improve walking distances, and improved walking distances are strongly correlated with improved skeletal muscle mass and resting pain level [9,10]. Guidelines and meta-analysis recommended exercise therapy for PAD patients with claudication to improve their symptoms and manage the risk of cardiovascular events [1,8,11]. However, because exercise therapy for CLI may reduce tissue oxygen saturation in the ischemic limb, it is not recommended because little evidence is available. Persistent diminished peripheral perfusion in PAD patients causes increased adipose tissue in skeletal muscle and myocyte fibrosis, leading to skeletal muscle atrophy [12], which reduces motor function and leads to the progression of sarcopenia. Sarcopenia is associated with cardiovascular events in critical limb ischemia patients and may be a poor prognostic factor [13]. Exercise therapy not only improves peripheral blood flow by promoting angiogenesis but also increases the release of vasodilatory nitric oxide (NO) and endothelial nitric oxide synthase (eNOS) activity, thereby improving vascular endothelial function by alleviating oxidative stress [[14], [15], [16]]. Sustainable exercise also increases skeletal muscle mass and oral intake by improvement of activity, thereby maintaining strength and preventing sarcopenia, thus contributing to the prevention of arteriosclerosis progression by improving the lifestyle and helping to reduce cardiovascular events, which are the main cause of death for CLI patients.

Although supervised exercise therapy is recommended for PAD patients with claudication, exercise interventions for CLI patients have yet to be established because there is a risk that they may aggravate lower limb ischemia, and these patients are unable to benefit from exercise therapy. Therefore, we proposed a method of implementing exercise therapy using NIRS monitoring. NIRS enables the quantitative measurement of lower limb ischemia during and after exercise in terms of muscle tissue oxygen saturation [17]. Fitting the NIRS device to the leg and monitoring CLI patients during exercise therapy may enable the optimal amount of exercise necessary to avoid severe lower limb ischemia, meaning that they can safely undergo rehabilitation.

Establishing optimal exercise therapy for CLI patients may enable them to engage in exercise without severely diminishing tissue oxygen saturation, which could help improve the rates of both survival and limb salvage. Moreover, because blood vessel development occurs gradually over several months after therapeutic angiogenesis, its use in combination with exercise therapy may result in even greater angiogenesis.

It is hoped that optimal exercise therapy for CLI patients following therapeutic angiogenesis will encourage further angiogenesis, improve poor peripheral perfusion, avoid limb amputation, and improve resting pain. Improving the walking distance and increasing skeletal muscle may also help prevent sarcopenia, suppress cardiovascular events, and prevent the progression of arteriosclerosis, thereby potentially contributing to the survival prognosis of patients with CLI. Improving the walking distance is also expected to improve activities of daily living and quality of life, thereby potentially reducing the need for long-term care by CLI patients and increasing their opportunities for social participation.

## 4 Conclusions

Exercise therapy may improve the mental and physical functions of CLI patients as well as their lifestyles, and it may provide a practicable therapeutic option that enables total health care treatment. In addition to evaluating the safety and efficacy of optimal exercise therapy for CLI patients after therapeutic angiogenesis, we must also establish evidence for exercise therapy for these patients.

## Financial disclosure

This clinical trial was supported by Regenerative Medicine Commercialization Research Project from AMED under grant number JP19bk0104018.

## Declaration of competing interest

The authors declare no conflicts of interest associated with this trial.
